# A new oligonucleotide array for the detection of multidrug and extensively drug-resistance tuberculosis

**DOI:** 10.1038/s41598-019-39339-3

**Published:** 2019-03-14

**Authors:** Ching-Yu Chen, Jui-Yun Weng, Hsin-Hui Huang, Wen-Chun Yen, Yu-Han Tsai, Tsung Chain Cheng, Ruwen Jou

**Affiliations:** 1 Tuberculosis Research Center, Centers for Disease Control, Ministry of Health and Welfare, Taipei, 115 Taiwan; 2 Department of Medical Laboratory and Biotechnology, National Cheng Kung University, Tainan, 701 Taiwan; 3 Institute of Microbiology and Immunology, National Yang-Ming University, Taipei, 112 Taiwan

## Abstract

Drug-resistant tuberculosis (TB) is a global crisis and a threat to health security. Since conventional drug susceptibility testing (DST) takes several weeks, we herein described a molecular assay to rapidly identify multidrug-resistant (MDR) and extensively drug-resistant (XDR) and reveal transmission associated-mutations of *Mycobacterium tuberculosis* complex (MTBC) isolates in 6 to 7 hours. An array was designed with 12 pairs of primers and 60 single nucleotide polymorphisms of 9 genes: *rpoB*, *katG*, *inhA*, *ahpC*, *embB*, *rpsL*, *gyrA*, *rrs* and *eis*. We assessed the performance of the array using 176 clinical MTBC isolates. The results of culture-based DST were used as the gold standard, the GenoType MTBDR*plus* and MTBDR*sl* tests were used for parallel comparison, and gene sequencing was performed to resolve the discordance. The sensitivities and specificities of the array are comparable to those of the MTBDR*plus* test for resistance to isoniazid (INH) (100.0%, 96.7%) and rifampicin (RIF) (99.4%, 96.7%) and of the MTBDR*sl* test for resistance to fluoroquinolones (FQs) (100%, 100%) and second-line injectable drugs (SLIDs) (98.3%, 100%). The sensitivities of the array for detecting resistance to ethambutol and streptomycin were 79.3% and 64.9%, respectively. The array has potential as a powerful tool for clinical diagnosis and epidemiological investigations.

## Introduction

Drug-resistant tuberculosis (TB) is a public health concern and a threat to global TB control programs. The World Health Organization (WHO) estimated that there were 10 million new TB cases globally in 2017^1^. Moreover, an estimated 3.6% of new cases and 17% (15–28%) of previously treated cases were rifampicin (RIF)-resistant TB or multidrug-resistance (MDR) TB, which is defined as a *Mycobacterium tuberculosis* complex (MTBC) isolate that is resistant to at least RIF and isoniazid (INH)^1^. The combined regimen recommended for MDR-TB treatment should include a fluoroquinolone (FQ) or a second-line injectable drug (SLID), including kanamycin (KM), amikacin (AM), and capreomycin (CAP). MDR-TB cases can become extensively drug-resistant TB (XDR-TB), which is defined as MDR MTBC resistant to any FQ and at least one of the SLIDs. The WHO estimated that in 2017, 8.5% (6.2–11%) of MDR-TB cases were XDR-TB^1^.

The conventional culture-based first-line and subsequent second-line drug susceptibility testing (DST) usually requires 4–8 weeks to complete. Since drug-resistant TB is difficult to treat and has high mortality rate^2^, timely diagnosis of cases with molecular tests is crucial to improved management of drug-resistant TB. Drug-resistant MTBC is associated with mutations in several genes, including the *rpoB* gene for RIF; the *katG* gene, the *inhA* regulatory region and the *ahpC* gene for INH; the *emb B* gene for ethambutol (EMB); the *rpsL* and *rrs* genes for streptomycin (SM); *gyrA* and *gyrB* for FQs; and *rrs* and the promoter of *eis* for KM, AM and CAP. To accelerate the efficiency of drug-resistant TB diagnosis compared to that of the conventional cultured-based DST, several commercial tests and in-house molecular methods for identifying TB and drug resistance have been designed based on the aforementioned genes and adopted in clinical laboratories. Among the assays recommended by the WHO are the GeneXpert MTB/RIF (Cepheid, USA) assay, which detects MTBC and RIF resistance simultaneously from sputum and was recommended by the WHO in 2010, and 2 line-probe assays (LPAs), namely, the GenoType MTBDR*plus* v2.0 test (Hain Lifescience, Germany), which can be used to detect resistance to RIF and INH, and the GenoType MTBDR*sl* v2.0 test, which identifies resistance to FQs and SLIDs, and these two assays were also recommended by the WHO. The performance of the GenoType MTBDR*plus* and MTBDR*sl* tests are well documented^3–6^.

Multiplex PCR is widely developed for TB diagnosis. Regarding real-time multiplex PCR systems, Abbott RealTi*me* MTB INH/RIF Assays (Abbott, USA), as well as the Anyplex plus MTB/NTM and MDR-TB products (Seegene, Seoul, Korea), discriminate MTB or MDR-TB infection from respiratory specimens^7,8^. Array-based analysis of MTBC has also been shown to provide important information on the mechanism of drug resistance, the identity of the species or strain, and gene expression^9^. Commercial microarrays such as the BluePoint MycoID array^10^ are capable of identifying species of MTBC and non-tuberculous mycobacteria (NTM), whereas the DR. TBDR/NTM kit^11^ can distinguish MTBC from 15 species of NTM while detecting RIF resistance. Arrays that detect genetic resistance to first-line anti-TB drugs and second-line drugs have also been well studied^12–20^. The CapitalBio microarray has sensitivities of 88.9% and 80.0% for detecting RIF and INH resistance, respectively^21^. For the TB-Biochip microarray, the sensitivities for detecting RIF and INH resistance were 100% and 98.2%, respectively^22^. DNA arrays for detecting resistance to EMB^23^ or pyrazinamide (PZA)^15,20^ are also available. A low-density hydrogel microarray with sensitivities and specificities over 90% for RIF, INH, FQ (ofloxacin) and SLIDs resistance was reported, while its sensitivity and specificity for detecting EMB resistance were 89.9% and 57%, respectively^24^.

**Figure 1:**
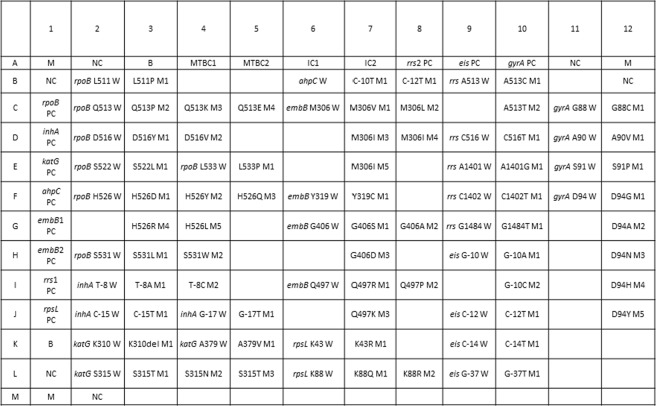
Layout of probes on the array. M, marker; B, baseline; PC, positive control; NC, negative control; IC, internal control; MTBC, *Mycobacterium tuberculosis* complex control; W, wild-type; M with number, mutation probe for different mutation polymorphism.

**Figure 2:**
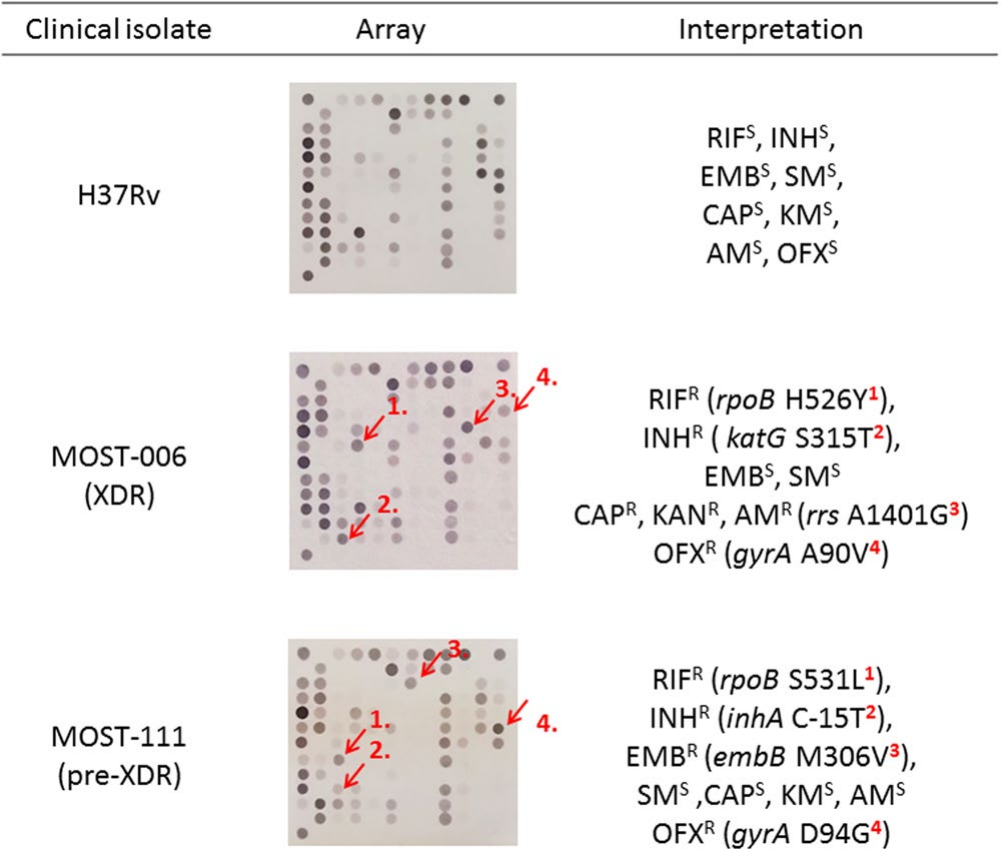
The hybridization patterns of a susceptible MTBC strain (H37Rv), an XDR strain (MOST-006), and a pre-XDR strain (MOST-111) analyzed by the array and result interpretations. XDR, extensively drug-resistant; RIF, rifampicin; INH, isoniazid; EMB, ethambutol; SM, streptomycin; CAP, capreomycin; KAN, kanamycin; AM, amikacin; OFX, ofloxacin; R, resistance; S, susceptible.

Previously, we developed and commercialized a nylon membrane array (BluePoint MtbDR, Bio Concept Inc., Taichung, Taiwan) for detecting gene mutations conferring resistance to RIF and INH in MTBC^25^. The BluePoint MtbDR array includes 17 probes for mutations in the *rpoB* hotspot region, 5 probes for codon 315 (*Escherichia coli rpoB* numbering and thereafter in this text) mutations of the *katG* gene, and 3 probes for the promoter region of the *inhA* gene. We herein designed a novel array that can detect resistance to 8 anti-TB drugs simultaneously by including probes that can identify gene mutations conferring RIF, INH, EMB, SM, FQ, and SLIDs resistance. Specifically, 60 probes were integrated into this array to detect single nucleotide polymorphisms (SNPs) of 9 drug resistance-related genes (Fig. 1). Examples of the drug resistance results obtained by the array are shown in Fig. 2. We report the performance of the new array and compare it with the performance of the GenoType MTBDR*plus* and GenoType MTBDR*sl* tests while using the conventional culture-based DST as a reference test.

## Results

### Characteristics of study isolates

The phenotypic DST results of 176 MTBC isolates are listed in Table 1. Of the 160 MDR MTBC isolates, 43 (24.4%) were simple MDR, 42 (22.5%) were pre-XDR-INJ, 36 (26.3%) were pre-XDR-FQ, and 39 (24.4%) were XDR. All 16 fully drug-susceptible MTBC isolates demonstrated WT sequences, as determined by the array and 2 LPAs. The frequencies of mutations detected by the array are listed in Tables 2–4.

**Table 1:** Phenotypic drug resistance profiles of 176 *Mycobacterium tuberculosis* complex isolates.

First-line drug resistance (RIF/INH/EMB/SM)	Second-line drug resistance (OFX/KM/AM/CAP)										
	RRRR	RRRS	RRSR	RRSS	RSSR	RSSS	SRRR	SRRS	SRSS	SSSS	Total
RRRR	9	10	2	3	2	23	7	3	5	13	77
RRRS	2				1	15	6	2	4	9	39
RRSR	4	2					1	2		8	17
RRSS	2	1	1			4	3	2	1	13	27
SSSS										16	16
Total	17	13	3	3	3	42	17	9	10	59	176

RIF, rifampicin; INH, isoniazid; EMB, ethambutol; SM, streptomycin; OFX, ofloxacin; KM, kanamycin; AM, amikacin; CAP, capreomycin; R, resistance; S, susceptible.

**Table 2:** Profiles of rifampicin and isoniazid resistance identified by phenotypic and molecular assays.

DST	Mutations detected by the array	GenoType MTBDR*plus*	Gene sequencing	No. of isolates (%)
Rifampicin				
S	WT	WT	—	15 (8.5)
R	*rpoB* L511P M1	∆ WT 2	—	1 (0.6)
R	*rpoB* Q513P M2	∆ WT 2	—	1 (0.6)
—	*rpoB* Q513K M3	—	—	0
—	*rpoB* Q513E M4	—	—	0
R	∆ *rpoB* 513 WT	∆ WT 4	Ins TTC at codon 514	1 (0.6)
R	*rpoB* D516Y M1	∆ WT 3,4	—	1 (0.6)
R	*rpoB* D516V M2	∆ WT 3,4	—	6 (3.4)
R	*rpoB* D516V M2	∆ WT 3,4,8	—	1 (0.6)
R	∆ *rpoB* 516 WT	∆ WT 3,4	—	1 (0.6)
R	*rpoB* S522L M1	∆ WT 5,6	—	1 (0.6)
R	*rpoB* H526D M1	∆ WT 7, MUT 2B	—	4 (2.2)
R	*rpoB* H526Y M2	∆ WT 7, MUT 2 A	—	15 (8.5)
—	*rpoB* H526Q M3	—	—	0
R	*rpoB* H526R M4	∆ WT 7	—	4 (2.2)
R	*rpoB* H526R M4	∆ WT 2,7	—	1 (0.6)
R	*rpoB* H526L M5	∆ WT 7	—	3 (1.7)
R	*rpoB* S531L M1	∆ WT 8, MUT 3	—	100 (56.8)
R	*rpoB* S531W M2	∆ WT 8	—	4 (2.2)
R	*rpoB* L533P M1	∆ WT 8	—	10 (5.7)
R	*rpoB* L533P M1	∆ WT 3,4,8	—	1 (0.6)
S	*rpoB* L533P M1	∆ WT 8	*rpoB* L533P	1 (0.6)*
R	*rpoB* L511P M1, H526D M1	∆ WT 2,7	—	1 (0.6)
R	*rpoB* D516Y M1, H526D M1	∆ WT 3,4,7	—	1 (0.6)
R	∆ *rpoB* 511, 513, 516 WT	∆ WT 2,3	*rpoB* L511P, D516G	1 (0.6)
R	∆ *rpoB* 511 WT	∆ WT 2,3,4	*rpoB* L511R, D516A	1 (0.6)*
R	*rpoB* WT	∆WT 7	*rpoB* R529K	1 (0.6)
Isoniazid				
S	WT	WT	—	16 (9.0)
R	WT	WT	WT	11 (6.3)*
R	WT	WT	*oxyR-ahpC* G-8A	1 (0.6)*
R	WT	WT	*oxyR-ahpC* C-17T	1 (0.6)*
—	*katG* K310del M1	—	—	0
R	*katG* S315T M1	∆ *katG* WT 1, MUT 1	—	78 (44.3)
R	*katG* S315N M2	∆ *katG* WT 1	—	2 (1.1)
R	*katG* S315T M5	∆ *katG* WT 1, MUT 2	—	1 (0.6)
—	*katG* A379V M1	—	—	0
R	*inhA* T-8A M1	∆ *inhA* WT 1, MUT 3B	—	1 (0.6)
R	*inhA* T-8C M2	∆ *inhA* WT 2, MUT 3 A	—	5 (2.8)
R	*inhA* C-15T M1	∆ *inhA* WT 1, MUT 1	—	39 (22.2)
—	*inhA* C-17T M1	—	—	0
R	*oxyR-ahpC* C-10T M1	WT	*oxyR-ahpC* C-10T	1 (0.6)*
—	*oxyR-ahpC* C-12T M2	—	—	0
R	*katG* S315T M1, *inhA* T-8A M1	∆ *katG* WT 1, MUT 1	—	2 (1.1)
R	*katG* S315 T M1, *inhA* C-15T M1	∆ *inhA* WT 1, MUT 3B	—	17 (9.7)
R	*inhA* C-15T, *oxyR-ahpC* C-10T	∆ *katG* WT 1, MUT 1	—	1 (0.6)*
S	WT	WT	—	16 (9.0)
R	WT	WT	WT	11 (6.3)*

R, resistant; S, susceptible; WT, wild-type; ∆, lack of wild-type signal; *discordant results between each assay.

**Table 3:** Profiles of ethambutol and streptomycin resistance identified by phenotypic and molecular assays.

DST	Mutations detected by the Array	Gene sequencing	No. of isolates (%)
Ethambutol			
S	WT	—	48 (27.3)
R	WT	WT	6 (3.4)*
R	WT	*embB* M306I (ATG/ATA)	1 (0.6)*
R	WT	*embB* Y319S	1 (0.6)*
R	WT	*embB* D328Y, Y334H	1 (0.6)*
R	WT	*embB* S347T, E504D	1 (0.6)*
R	WT	*embB* D354A	3 (1.7)*
R	WT	*embB* L355L, E378A, A505V	6 (3.4)*
R	WT	*embB* C361Y	1 (0.6)*
R	WT	*embB* N399T	1 (0.6)*
R	WT	*embB* G406A	1 (0.6)*
R	WT	*embB* Q497R, S565G	1 (0.6)*
R	WT	*embB* D534D	1 (0.6)*
R	*embB* M306V M1	—	36 (20.5)
S	*embB* M306V M1	*embB* M306V	2 (1.1)*
R	*embB* M306L M2	—	5 (2.8)
R	*embB* M306I M3	—	15 (8.5)
R	*embB* M306I M4	—	2 (1.1)
S	*embB* M306I M4	*embB* M306I (ATG/ATT)	1 (0.6)*
R	*embB* M306I M5	—	6 (3.4)
S	*embB* M306I M5	*embB* M306I (ATG/ATA)	3 (1.7)*
R	*embB* Y319C M1	—	1 (0.6)
S	*embB* Y319C M1	*embB* Y319C	1 (0.6)*
R	*embB* G406S M1	—	1 (0.6)
S	*embB* G406S M1	*embB* G406S	1 (0.6)*
R	*embB* G406A M2	—	1 (0.6)
S	*embB* G406A M2	*embB* G406A	1 (0.6)*
R	*embB* G406D M3	—	1 (0.6)
R	*embB* Q497R M1	—	6 (3.4)
R	*embB* Q497P M2	—	14 (8.0)
S	*embB* Q497P M2	*embB* Q497P	1 (0.6)*
R	*embB* Q497K M3	—	3 (1.7)
R	*embB* M306I M3, Q497K M3	—	1 (0.6)
R	*embB* M306I M5, G406D M3	—	1 (0.6)
R	*embB* M306I M5, Q497R M1	—	1 (0.6)
Streptomycin			
S	WT	—	77 (43.8)
R	WT	WT	28 (18.8)*
R	WT	*rpsL* K88R	5 (2.8)*
R	*rpsL* K43R M1	*rpsL* K43R	46 (26.0)
S	*rpsL* K43R M1	*rpsL* K43R	2 (1.1)*
—	*rpsL* K88Q M1	—	0
R	*rpsL* K88R M2	—	4 (2.3)
R	*rrs* A513 C M1	—	10 (5.7)
S	*rrs* A513C M1	*rrs* A513C	1 (0.6)*
R	*rrs* A513T M2	—	1 (0.6)
R	*rrs* C516T M1	—	1 (0.6)
S	*rrs* C516T M1	*rrs* C516T	2 (1.1)*

R, resistant; S, susceptible; WT, wild-type; ∆, lack of wild-type signal; *discordant results between each assay.

**Table 4:** Profiles of fluoroquinolone and second-line injectable drug resistance identified by phenotypic and molecular assays.

DST	Mutations detected by the Array	GenoType MTBDR*sl*	Gene sequencing	No. of isolate (%)
FQ				
S	WT	WT	—	94 (53.4)
R	WT	WT	WT	4 (2.3)*
—	*gyrA* G88C M1	—	—	0
R	*gyrA* A90V M1	∆ *gyrA* WT 2	—	16 (9.1)
R	*gyrA* S91P M1	∆ *gyrA* WT 2	—	3 (1.7)
S	*gyrA* S91P M1	∆ *gyrA* WT 2	*gyrA* S91P	1 (0.6)*
R	*gyrA* D94G M1	∆ *gyrA* WT 3, MUT 3C	—	32 (18.2)
R	*gyrA* D94A M2	∆ *gyrA* WT 3, MUT 3 A	—	4 (2.3)
R	*gyrA* D94N M3	∆ *gyrA* WT 3, MUT 3B	—	17 (9.7)
R	*gyrA* D94H M4	∆ *gyrA* WT 3, MUT 3D	—	2 (1.1)
R	*gyrA* D94Y M5	∆ *gyrA* WT 3	—	3 (1.7)
KM/AM/CAP				
SSS	WT	WT	—	99 (56.3)
SSR	WT	WT	WT	2 (1.1)*
RRR	*rrs* A1401G M1	∆ *rrs* WT 1, MUT 1	—	32 (18.2)
RRS	*rrs* A1401G M1	∆ *rrs* WT 1, MUT 1	*rrs* A1401G	21 (11.9)*
RSR	*rrs* C1402T M1	∆ *rrs* WT 1	—	2 (1.1)
RRR	*rrs* G1484T M1	∆ *rrs* WT 2, MUT 2	—	2 (1.1)
SRS	*eis* G-10A M1	∆ *eis* WT 2	—	5 (2.8)
—	*eis* G-10C M2	—	—	0
RSS	*eis* C-12T M1	∆ *eis* WT 2	—	2 (1.1)
SSR	*eis* C-12T M1	∆ *eis* WT 2	*eis* C-12T	1 (0.6)*
RSS	*eis* C-14T M1	∆ *eis* WT 2, MUT 1	—	3 (1.7)
RSR	*eis* C-14T M1	∆ *eis* WT 2, MUT 1	—	1 (0.6)
RRS	*eis* C-14T M1	∆ *eis* WT 2, MUT 1	—	1 (0.6)
RSS	*eis* G-37T M1	*eis* MUT 1	*eis* G-37T/ WT	1 (0.6)*
RSS	*eis* G-37T M1	∆ *eis* WT 1	—	2 (1.1)

R, resistant; S, susceptible; WT, wild-type; ∆, lack of wild-type signal; *discordant results between each assay.

### Detection of drug resistance with the array

As an example of the interpretation of the final results obtained from the array, Fig. 2 shows the detection of H37Rv DNA and 2 isolates with drug resistance using the array. Excluding one of the *embB* probe signals, the detection limit of the array was 300 pg per test. Compared with conventional DST, the sensitivities of the array for detecting resistance to the 8 drugs were over 90%, except for those of EMB and SM, which were 79.3% and 64.9%, respectively. The specificities of the array ranged from 80.0% to 100% (Table 5).

**Table 5:** Performance of the array in detecting resistance to 8 anti-tuberculosis drugs.

Drugs	Parameter		
	No. of isolates (R/S)	Sensitivity (%) [95% CI]	Specificity (%) [95% CI]
Rifampicin	160/16	98.8 (95.6–99.9)	93.8 (69.8–99.8)
Isoniazid	160/16	91.3 (85.8–95.1)	100.0 (79.4–100)
Ethambutol	116/60	79.3 (70.8–86.3)	80.0 (67.7–89.2)
Streptomycin	94/82	64.9 (54.4–74.5)	93.9 (86.3–98.0)
Ofloxacin	81/95	95.1 (87.8–98.6)	98.9 (94.3–100)
Kanamycin	72/104	100.0 (95.0–100)	98.1 (93.2–99.8)
Amikacin	56/120	98.2 (90.5–100)	98.3 (94.1–99.8)
Capreomycin	40/136	90.0 (76.3–97.2)	84.6 (77.4–90.2)

CI, confidence interval; R, resistance; S, susceptible.

We evaluated the sensitivity and specificity of the array relative to those of the GenoType MTBDR*plus* v2.0 and MTBDR*sl* v2.0 tests. Compared to both GenoType assays, the sensitivities of the array were 99.4% (95% CI 96.6–99.9) for detecting resistance to RIF, 100% (95% CI 97.5–100) for detecting resistance to INH, 98.3% (95% CI 90.8–99.9) for detecting resistance to SLIDs and 100% (95% CI 95.4–100) for detecting resistance to FQs. The specificities of the array were comparable to those of the GenoType MTBDR*plus* v2.0 and MTBDR*sl* v2.0 tests, with 100% (95% CI 79.4–100) for detecting resistance to RIF, 96.7% (95% CI 82.8–99.9) for detecting resistance to INH, 100% (95% CI 96.9–100) to for detecting resistance SLIDs and 100% (95% CI 96.4–100) for detecting resistance to FQs. We also observed good agreement between the array and the GenoType tests regarding the detection of resistance to RIF, INH, SLIDs and FQ with kappa values of 0.97, 0.98, 0.99 and 1, respectively. There was no statistically significant difference between the array and the GenoType tests (Table 6).

**Table 6:** Drug resistance in 176 *Mycobacterium tuberculosis* complex isolates detected using the array and the GenoType MTBDR*plus* and MTBDR*sl* tests.

Drugs	Array	MTBDR*plus*/*sl* v2.0		Sensitivity (%) [95% CI]	Specificity (%) [95% CI]	Kappa value	P value
		R	S				
Rifampicin	R	159	0	99.4 (96.6–99.9)	100 (79.4–100)	0.97	1
	S	1	16				
Isoniazid	R	146	1	100 (97.5–100)	96.7 (82.8–99.9)	0.98	1
	S	0	29				
Fluoroquinolone	R	78	0	100 (95.4–100)	100 (96.4–100)	1	
	S	0	98				
Second-line injectable drugs	R	57	0	98.3 (90.8–99.9)	100 (96.9–100)	0.99	1
	S	1	118				

CI, confidence interval.

### Analysis of divergent results between the molecular assays and phenotypic DST

For detecting RIF resistance, we obtained divergent results with 1.8% (3/176) of the isolates. One of these three was a phenotypic RIF-susceptible isolate that lacked WT bands 2, 3 and 4 on the RIF-determining region of the GenoType MTBDR*plus* strip and the WT signal of the *rpoB* codon 511 when using the array. *rpoB* gene sequencing revealed the presence of the *rpoB* D516A mutation, which was not included in the array (Fig. 1). Of the 2 phenotypic RIF-resistant isolates, one isolate harbored two mutations, *rpoB* L511P and D516G, as revealed using *rpoB* gene sequencing; conversely, the array showed the WT signal. The partial overlap between the sequences of the probes for detecting codons 511 and 513 may cause a false negative. The other isolate had the *rpoB* R529K mutation, which was not included in the array but was correctly identified by the GenoType MTBDR*plus* test. In addition, a fully susceptible isolate with the *rpoB* gene L533P mutation was detected by the array, the GenoType MTBDR*plus* test and gene sequencing (Table 2). For INH-resistance detection, we found discordant results with 8.5% (15/176) of the isolates. The array and the GenoType MTBDR*plus* test provided WT results for 6.3% (13/160) of the phenotypic INH-resistant isolates (Table 2). The results from sequencing *katG*, the *inhA* locus and the *oxyR-ahpC* intergenic region revealed that among these isolates, only 2 harbored mutations in the *oxyR-ahpC* intergenic region, G-8A and C-17T, which were not included in the array or the GenoType MTBDR*plus* test. One isolate harbored the C-10T mutation in the *oxyR-ahpC* intergenic region according to the array and sequencing, but this mutation was missed by the GenoType MTBDR*plus* test. The other isolate showed C-15T mutations in the *inhA* locus and the C-10T mutation in the *oxyR-ahpC* intergenic region (Table 2).

For detecting EMB resistance, we found that 19.3% (34/176) of the isolates had discordant results. In total, 20.7% (24/116) of the phenotypic EBM-resistance isolates were deemed to be susceptible by the array. *embB* gene sequencing revealed *embB* Y319S, D328Y, Y334H, S347T, D354A, C361Y, N399T and S565G mutations, which did not have corresponding probes incorporated into the array. However, 16.7% (10/60) of the phenotypic EMB-susceptible strains harbored *embB* M306I, Y319C, G406S, G406A and Q497P mutations (Table 3).

For detecting SM resistance, we found that 18.8% (33/176) of the isolates afforded divergent results. The array and *rpsL* and *rrs* gene sequencing identified 84.8% (28/33) of the phenotypic SM-resistant isolates as SM susceptible. Moreover, *rpsL* gene sequencing revealed 5 isolates with *rpsL* codon K88R mutation, but this was missed by the array (Table 5). Nevertheless, we found that 6% (5/82) of the SM-susceptible isolates harbored *rpsL* k43R (n = 2), *rrs* A513C (n = 1) and *rrs* C516T (n = 2) mutations, as determined by the array and gene sequencing (Table 3).

For detecting FQ resistance, we obtained discordant results for 2.8% (5/176) of the isolates. In total, 4.9% (4/81) of the phenotypic FQ-resistant isolates were identified as FQ susceptible by the array, the GenoType MTBDR*sl* test and *gyrA* gene sequencing, with 0.1% (1/95) of the phenotypic FQ-susceptible isolate harboring the *gyrA* S91P mutation (Table 4).

For detecting SLID resistance, we found that 14.2% (25/176) isolates afforded divergent results. Of the 75 phenotypic SLID-resistant isolates, 2 (2.6%) CAP-resistant isolates were identified as susceptible by the array, the GenoType MTBDR*sl* test and *rrs* gene sequencing. Furthermore, 21 (28%) isolates with the *rrs* A1401G mutation detected by all three molecular methods were deemed susceptible by phenotypic DST. Two isolates that harbored the *eis* C-12T and G-37T mutations respectively, as detected by the 3 molecular methods, were identified as phenotypic KM susceptible. In addition, one fully SLID-susceptible isolate revealed that deletion at *eis* -8 was not identified by the array; however, this isolate was correctly identified by the GenoType MTBDR*sl* test as lacking *eis* WT2.

## Discussion

Because of the urgent need to rapidly and accurately diagnose drug-resistant TB, the WHO has recommended the GenoType MTBDRplus and GenoType MTBDRsl tests for detecting resistance to first- and second-line drugs^1^. We developed an oligonucleotide array consisting of 60 mutant probes for detecting resistance to 8 anti-TB drugs in 6 to 7 hours. The sensitivity and specificity of the array were comparable to those of the WHO-recommended GenoType MTBDRplus (91.3% and 98.0% for RIF; 89.4% and 98.9 for INH), the GenoType MTBDR*sl* tests (94.8% and 98.% for FQs; 83.0–91.3% and 94.3–100% for SLIDs), the AID TB resistance LPA (AID Diagnostika, Germany) (100% and 100% for RIF; 97.8% and 100% for INH; 60.0% and 91.7% for EMB; 100% and 96.6% for SM; 33.3% and 98.1% for FQs; 100% and 100% for SLIDs), the Nipro NTM/MDRTB detection kit (Nipro, Japan) (92.4% and 97.5% for RIF; 89.9% and 99.4% for INH) and the Xpert MTB/RIF Ultra (Cepheid, USA)(92.7% and 98.0% for RIF)^6,26–29^. Moreover, our array contains multiple mutation probes targeting the same alleles as the other aforementioned tests to achieve better sensitivity and accuracy.

This array can identify exact nucleotide substitutions in the mutated codon(s) to provide a clearer interpretation than those LPAs with fewer mutation probes. In addition, our system can simultaneously determine first- and second-line drug resistance in a single array, whereas the above commercial LPAs detect resistance to first- and second-line drug in separate strips, which may delay the turnaround time for determining the genotypic susceptibility to second-line drugs when cases are identified as RIF-resistant or MDR-TB. Furthermore, the expenditure for multi-step diagnosis is a huge burden for low- to middle-income countries.

The high concordance for detecting RIF resistance was expected because over 95% of RIF-resistant strains harbor mutations in the rifampicin-resistance determining (RRDR) region^30,31^. The one discordant profile obtained from phenotypic and molecular assays had the* rpoB* L533P mutation, which confers low-level resistance to RIF^32,33^. Low-level drug resistance is a major challenge in clinical diagnosis, especially for those laboratories that use the Mycobacteria growth indicator tube (MGIT) as the phenotypic assay^34^.

Concerning INH resistance, eleven INH-resistant strains were missed by the array. Thus, we considered potentially including other mutations of resistance-related gene in our bacteria set, such as the *inhA* structural gene specifically, the S94A and I194T mutations, which have been reported to be associated with high-level resistance to both INH and ethionamide in MDR *M*. *tuberculosis* strains identified in Latin American-Mediterranean families in Lisbon^35^. The sensitivity and specificity of the array for detecting INH resistance were comparable to those of GenoType MTBDR*plus*, which were 100% and 96.7%, respectively. We also designed two specific probes for detecting mutations in the *oxyR-ahpC* intergenic region, i.e., C-10T and C-12T, which are absent from the INH-resistance determining region of the GenoType MTBDR*plus* test. Accordingly, the array can correctly identify resistant isolates in agreement with the sequencing results. However, the G-8A and C-17T substitutions in the *oxyR-ahpC* intergenic region, which were found by gene sequencing, were not included in our probe panel; thus, we perhaps should consider these mutations as alternatives for detecting INH resistance.

We observed low sensitivity for detecting EMB resistance, which might be due to the presence of mutations in the *embA* and *embC* genes^36^. In addition, some rare resistance-related mutations that are identified by gene sequencing, such as the *embB* codon Y319S mutation, were also not included in the array. Three mutations corresponding to EMB resistance were missed by array: the *embB* codon M306I, G406A and Q497R mutations. We re-checked the raw data of the array for these three isolates, which revealed that the signal of the mutation probes of the aforementioned three locations, were too weak to identify, causing us to misjudge the output at first. The main reason for weak signals include low yield of *embB* PCR products, low melting temperature of the probe, probe length, probe self-binding and the formation of secondary structures within the PCR products. All probes designed in this study were checked for internal repeat, self-binding, secondary structure, and GC content by using software Vector NTI (Invitrogen Corporation, Carlsbad, Cal. USA). However, the software may not be able to reveal all drawbacks inherently present in the probes and therefore probe efficacy cannot be guaranteed. In this study, normally 5–10 probes were designed, checked by software, pretested by array hybridization, and finally the one with the best hybridization signal was used in the current array. Longer probes can have a stronger hybridization signal, but at the same time, longer probes may decrease the specificity of the probes. Therefore, a probe used in the array is a compromise of sensitivity and specificity. Conversely, six isolates showed the same genetic profile with triple mutations of *embB* L355L, E378A and A505V; we reviewed the registry of all six cases and found that it was an outbreak within a family. The selection bias was due to the selection of drug-resistant strains according to only phenotypic DST without initially confirming the clinical data.

Concerning SM, 28 of 33 SM-resistant isolates were identified as sensitive by the array and sequencing; the remaining 5 isolates carrying the *rpsL* K88R mutation as revealed by gene sequencing were missed by the array (Table 5). The 5 missing results were mainly due to the same reason mentioned above for EMB. The discordant phenotypic/genotypic profile harbored the *rpsL* K43R, *rrs* A513C and *rrs* C516T mutations, which were found in 5 SM-susceptible isolates. In various studies, *rpsL* and *rrs* mutations were found in 36.6% to over 90% of SM-resistant isolates^37–40^. Additionally, recent studies indicated that the *gidB* gene encoding a 7-methylguanosine (m7G) methyltransferase specific to the 16 S rRNA was associated with low-level SM resistance in *M*. *tuberculosis*^41,42^. To improve the array performance in detecting SM, we might need to consider including some specific primers and probes for detecting the *gidB* gene.

With regard to FQs, the array results were 100% consistent with those from the GenoType MTBDR*sl* test. However, 4 FQ-resistant isolates were identified as FQ susceptible by the array, GenoType *sl* test and sequencing. Furthermore, we found one phenotypic FQ-susceptible isolate harboring the *gyrA* S91P mutation, as identified by the array, the GenoType MTBDR*sl* test and sequencing. The *gyrA* S91P mutation confers moderate- to low-level FQ resistance^43^.

Regarding SLIDs, two CAP-resistant isolates were identified as susceptible by the array, the GenoType MTBDR*sl* test and sequencing. The discordant results may be due to the involvement of other resistance-related genes, such as *tlyA*^44^. Nevertheless, 21 phenotypic susceptible strains containing the *rrs* A1401G mutation were correctly identified by the array, the GenoType MTBDR*sl* test and sequencing. On the one hand, some studies found that the *rrs* A1401G mutation confers high-level resistance to KM and AM but only low-level CAP resistance^45,46^, which suggests that CAP is still an alternative option for combination chemotherapy^47,48^. On the other hand, some discordant results were found in 3 isolates with *eis* mutations, i.e., deletion at -8, C-12T and G-37T. The substitution of *eis*-8 was not detected by our detection probes but was correctly identified by the GenoType MTBDR*sl* test, as WT band deletion in the GenoType strip should be considered indicative of resistance. The mutations within the *eis* promoter region conferring KM resistance still need to be confirmed. Several studies indicated that the -10 or -12 substitution has very little or no association with resistance to KM^49,50^.

Nucleic acid amplification tests (NAATs) are widely used in clinical diagnosis, such as real-time PCR assays (e.g., the GeneXpert MTB/RIF test), LPAs (e.g., the GenoType MTBDR*plus* test), and reverse hybridization microarrays (e.g., the BluePoint MtbDR array). DNA concentration is the principal limitation of the multiplex PCR assay; when the DNA concentration is inadequate for performing amplification, false negatives and low sensitivity may result. Furthermore, DNA quantification could play an important role in TB clinical management for characterizing the disease, transmission, and response to therapy^51^. In our study, the signals from the probes for *embB* and *rpsL* were too weak for some isolates, preventing us from making a correct identification. The misjudgments were probably due to low DNA concentration. In addition, the probes or PCR products of interest may form a secondary structure and hence decrease the hybridization efficiency^52^. Thus, preventing the formation of secondary structure of the probes and PCR products, which significantly decreases the detection signal, may become a challenging problem for multiplex PCR in a single tube.

Real-time PCR assays such as the GeneXpert MTB/RIF test, the Abbott RealTi*me* MTB INH/RIF Assays (Abbott, USA) and the Anyplex plus MTB/NTM MDR-TB products (Seegene, South Korea) are extensively used to diagnose TB with high accuracy and a fast turnaround time^53,54^. Although array hybridization takes multiple steps, other assays require an expensive instrument for detecting fluorescent signals and/or high-cost cartridges. In contrast, the cost of the array is approximately 10 to 15 US dollars, almost half the price of one GeneXpert MTB/RIF test^55^. Other than cost, no expensive equipment is required and the logistics are much simpler than either the Xpert test (imported with *in vitro* diagnostics approval by Taiwan Food and Drug Administration) or the 2 LPAs (imported without *in vitro* diagnostics approval by Taiwan Food and Drug Administration) assays. The only instrument used in this study is a small incubator with a shaking function for performing the hybridization process, rendering the array an affordable way to routinely conduct molecular surveillance in a high burden area. Compared to the Xpert test, the array could detect 8 drugs in a single assay. Compared to the LPA, when testing for XDR-TB, one will need to perform 2 LPAs, which is time-consuming and costly. Besides, our array could detect the exact mutations conferring drug resistance and avoid the detection of false RIF resistance, such as L511P by the Xpert test and the GenoTypeDR*plus* test. Furthermore, we could incorporate other probes to other resistance alleles into the array to enhance the performance, or we can remove some probes (incidental polymorphisms unrelated to resistance) with much lower detection rates than the others to improve the hybridization signal.

In conclusion, the results of an oligonucleotide array strongly agreed with those obtained using 2 WHO-recommended GenoType tests. The array is suitable for detecting MDR or XDR-TB and is a useful tool for epidemiological investigations with a short turnaround time. The array can have a significant impact on treating patients and on preventing the spread of drug-resistant TB.

## Methods

### *Mycobacterium tuberculosis* complex isolates

To ensure the genetic diversities of the drug-resistant MTBC isolates, we selected 176 MTBC isolates with diverse genotypes and drug resistance profiles from authorized clinical TB laboratories throughout Taiwan. The challenge MTBC set contained 3 main lineages including 16 lineage-1 strains (13 EAI2-Malina, 1 EAI1-SOM, 1 EAI2 and 1 EAI-5), 110 lineage-2 strains (96 Beijing ST1, 1 ST190, 1 ST250, 1 ST941; 7 Manu_ancestor 523 and 4 unknown ST955), 29 lineage-4 strains (4 H ST742, 1 ST946; 12 H3 ST50, 1 ST293, 1 ST316, 1 ST390, 1 ST511, 1 ST871, 1 ST2086, 1 ST2090, 1 ST 2091; 2 T1 ST53, 1 ST756, 1 ST1060; 1 T2 ST2699), 1 BOVIS ST684 and 1 ambiguous T3 ST73, 17 undefined and 2 unknown (1 ST 1487 and 1 ST2587).

### Drug susceptibility testing

MTBC isolates were subjected to DST using the proportion method with 7H10 medium (Becton, Dickinson and Company, Spark, MD, USA). Resistance was defined as 1% of the colonies growing in the presence of the following critical concentrations of first-line drugs: INH, 0.2 μg/ml; RIF, 1 μg/ml; SM, 2 μg/ml; and EMB, 5 μg/ml; and second-line drugs: AM, 6 μg/ml; KM, 6 μg/ml; CAP, 10 μg/ml; and ofloxacin (OFX), 2 μg/ml^25^. Growth on the control medium was compared to the growth on the drug-containing medium to determine susceptibility. The DST results were categorized as resistant or susceptible. The tests were validated by determining the susceptibility of *M*. *tuberculosis* H37Rv. MDR-TB is defined as an *M*. *tuberculosis* isolate that is resistant to at least INH and RIF. XDR-TB is defined as an MDR MTBC isolate that is resistant to at least one FQ and one SLID, whereas pre-XDR-TB is defined as an MDR*-*TB isolate that is resistant to either FQs (pre-XDR-FQ) or at least one of the injectable drugs (pre-XDR-INJ).

### Molecular assays

DNA was prepared according to the protocol provided by the manufacturer of the GenoType MTBDR*plus* v2.0 and MTBDR*sl* v2.0 kits and used for the array and DNA sequencing. The LPA assays were performed and results were read according to the manufacturer’s instructions.

The layout of the probes in our array is illustrated in Fig. 1. The array can be used in a biosafety level 1 laboratory. Our array hybridization procedures consisted of the amplification of the regions containing 9 genes using a multiplex PCR. The digoxigenin-labeled amplicons were hybridized with probes immobilized on the array. Most reagents, except buffers, were included in the DIG Nucleic Acid Detection kit (Roche, Cat. No. 1175041). The protocol used for array hybridization was as follows: 20 ml of 0.5 × SSC [(1 × SSC is 0.15 M NaCl, 0.015 M sodium citrate) plus 0.1% sodium dodecyl sulfate (SDS)] wash buffer was added to a 9-cm Petri dish containing the arrays for 10 min at room temperature, and the wash buffer was subsequently discarded. Each individual array was placed into a 24-well cell culture plate, and 300 μl of hybridization buffer [5× SSC, 1% (w/v) blocking reagent, 0.1% N-laurylsarcosine and 0.02% SDS] was added to each well. PCR products obtained by multiplex were denatured at 99 °C for 5 min and put on ice immediately. Hybridization was performed by adding 20 μl of digoxigenin-labeled PCR products into each well and incubating at 50 °C and 150 rpm for 90 min. After hybridization, all the following procedures were performed at room temperature. The arrays were washed twice with 0.5 × SSC plus 0.1% SDS for 5 min and were incubated with 20 ml of 1 × blocking solution [1% (w/v) blocking reagent dissolved in maleic acid buffer (0.1 M maleic acid, 0.15 M NaCl, pH 7.5)] containing 8 μl diluted 1:2500 alkaline phosphatase-conjugated sheep anti-digoxigenin antibodies (Roche, Cat. No. 11093274910) for 1 hour at 150 rpm. The antibody solution was removed, and then the arrays were washed with 20 ml of maleic acid buffer (0.1 M maleic acid, 0.15 M NaCl, pH 7.5) and 20 ml of detection buffer (0.1 M Tris-HCl, 0.15 M NaCl, pH 9.5). Color development of each array was achieved by incubation with 75 μl of alkaline phosphatase substrate (stock solution of nitroblue tetrazolium chloride/5-bromo-4-chloro-3-indolylphosphate diluted 1:50 in detection buffer) for 15 min away from light. After color development, the arrays were washed twice with distilled water for 5 min each time and dried out in an oven. Drug resistance was established when the hybridization signal of the mutation probe was stronger than that of the corresponding wild-type (WT) probes.

### Determination of detection limit of the array

Genomic DNA of *M*. *tuberculosis* H37Rv was used to determine the detection limit of the array. The DNA was serially diluted 10-fold (1 ng/μl to 100 fg/μl) with a carrier DNA (polyadenylic acid, Sigma-Aldrich, Saint Louis, MO, USA; 1 μg/ml in phosphate-buffered saline). The diluted DNA (2.5 μl) was used for PCR and then hybridized to the array. The detection limit of the array was defined as the highest dilution with signal that could be identified.

### Discordance analysis

DNA sequencing was performed by the Sanger method to resolve the divergent results between the array, the GenoType MTBDR*plus* v2.0 and MTBDR*sl* v2.0 tests, and phenotypic DST. DNA sequencing targeted mutations in *rpoB*, the *inhA* locus, *katG*, the *oxyR-ahpC* intergenic region, *embB*, *rrs*, *rpsL*, the *eis* promoter region, and *gyrA*. These regions were amplified by PCR and then further sequenced as previously described to detect mutations^25^.

### Data analysis

The sensitivity, specificity and agreement of the molecular assays were calculated using phenotypic DST as the gold standard. The degree of agreement between the array and the GenoType tests was assessed using the kappa statistic. Values of the kappa coefficient over 0.75 indicated excellent agreement, 0.40 to 0.75 as fair to good, and below 0.40 as poor. Statistical significance was evaluated by the McNemar test from QuickCalcs (https://www.graphpad.com/quickcalcs/McNemar1.cfm), and the two-tailed p < 0.05 was considered statistically significant.

### Ethical statement

This study was approved by the Institutional Review Board of Centers for Disease Control, Ministry of Health and Welfare (TwCDC IRB No. 103114). The study analyzed only archived *M*. *tuberculosis* isolates, and written informed consent of the participants was waived. All methods were performed in accordance with the relevant guidelines and regulations.
